# Drone-Based Position Detection in Sports—Validation and Applications

**DOI:** 10.3389/fphys.2022.850512

**Published:** 2022-03-17

**Authors:** Tiago Guedes Russomanno, Patrick Blauberger, Otto Kolbinger, Hilary Lam, Marc Schmid, Martin Lames

**Affiliations:** ^1^ Chair of Performance Analysis and Sports Informatics, Department of Sport and Health Sciences, Technical University of Munich, Munich, Germany; ^2^ Laboratory for Teaching Computer Science Applied to Physical Education and Sport, Faculty of Physical Education, University of Brasilia, Brasilia, Brazil

**Keywords:** drone, video based, position detection, game sport, validation

## Abstract

Radio and video-based electronic performance and tracking systems (EPTS) for position detection are widely used in a variety of sports. In this paper, the authors introduce an innovative approach to video-based tracking that uses a single camera attached to a drone to capture an area of interest from a bird’s eye view. This pilot validation study showcases several applications of this novel approach for the analysis of game and racket sports. To this end, the authors compared positional data retrieved from video footage recorded using a drone with positional data obtained from established radio-based systems in three different setups: a tennis match during training with the drone hovering at a height of 27 m, a small-sided soccer game with the drone at a height of 50 m, and an Ultimate Frisbee match with the drone at a height of 85 m. For each type of playing surface, clay (tennis) and grass (soccer and Ultimate), the drone-based system demonstrated acceptable static accuracy with root mean square errors of 0.02 m (clay) and 0.15 m (grass). The total distance measured using the drone-based system showed an absolute difference of 2.78% in Ultimate and 2.36% in soccer, when compared to an established GPS system and an absolute difference of 2.68% in tennis, when compared to a state-of-the-art LPS. The overall ICC value for consistency was 0.998. Further applications of a drone-based EPTS and the collected positional data in the context of performance analysis are discussed. Based on the findings of this pilot validation study, we conclude that drone-based position detection could serve as a promising alternative to existing EPTS but would benefit from further comparisons in dynamic settings and across different sports.

## 1 Introduction

Metrics generated by the different position detection technologies are already commonplace for fans of elite sports such as soccer, rugby, basketball, and American football (Barbon Junior et al., 2021; Schmid et al., 2021; Blair et al., 2022; Charamis et al., 2022). Currently, video-based systems that use image recognition are popular for live sports broadcasts and rely on several fixed cameras set up around the field of play. However, there are a number of constraints regarding the location of these systems. For example, the cameras must be placed at a sufficient height, which is often only possible in stadia and other well-equipped training facilities (Siegle et al., 2013; Torres-Ronda et al., 2022).

Besides, previous studies have found that video-based electronic performance and tracking systems (EPTS) for outdoor sports share some limitations, like occlusion during corner kicks in soccer (Iwase and Saito, 2004; Qi et al., 2004; Baysal and Duygulu, 2016; Kim et al., 2018), that can only be overcome by human corrections or the use of more cameras, consequently increasing the cost. Another point is associated with the use of fixed cameras versus moving cameras, as this can vary the complexity of the player tracking process (Chen et al., 2013; Hanzra and Rossi, 2013). Although it is possible, in principle, to use moving (tilting, swaying, zooming) cameras, almost all commercial systems work with fixed cameras.

With position detection becoming increasingly popular, many sport clubs have adopted sensor-based EPTS [e.g (Global position system (GPS)/Global Navigation Satellite System (GNSS)- or radio-based systems/local based system (LPS)], since most training facilities are not suitable for the installation of video-based EPTS. Sensor-based systems are also less costly than video-based systems, which would make it possible for amateur or minor league sports to use this technology. It is important to notice that the use of sensor based EPTS in training is not always possible in stadia as pointed by Shergill et al. (2021), that analyzed the quality of the signal during professional football matches and found out that the position of the players affected the quality of the GNSS signal and therefore their performance measurements.

Nevertheless, the diversity of EPTS available on the market poses a challenge for game analysts, as these different sources of data are typically incompatible. Consequently, comparisons of the players’ performances between these different systems, is difficult. This issue has been reported in the literature (Varley et al., 2012; Buchheit et al., 2014; Ellens et al., 2021). This incompatibility and the lack of interchangeability between systems creates a need for a single system capable of providing position detection data in both competition and training settings.

It would be a unique and worthwhile advancement for performance analysis if there was an affordable and reliable EPTS for teams and sport associations with small budgets that could collect data independent from setting, different stadia, or training sites. Unmanned aerial vehicles (UAV), commonly known as drones, could be the solution. Drones are widely available in the consumer market and have been used for several different applications so far, such as agriculture, surveillance, cinematography, and in some cases, during sports events to enhance the spectator experience (Ayranci, 2017). In recent years, drone technology for consumers has advanced so much that relatively inexpensive devices with decent flight characteristics are available and from which high-quality video recordings can be made. So, potentially, drones could play an important role in future in performance analysis. Compared to fixed cameras, these devices are portable and versatile, offer an aerial perspective of the playing field, and produce high quality videos that are suitable for broadcasting, position detection, and tactical analysis. With the ability to analyze the playing field with a single camera and without the need to install any equipment, performance analysts could consider using drones as an alternative to the existing video-based and sensor-based position detection technologies.

Regarding the positional data that can be obtained from drone footage, a review of the literature shows that several different methods based on image processing and computer vision have been used to automatically track players in a variety of sports (Cai and Aggarwal, 1996; Araki et al., 2000; Pers and Kovačič, 2000; Needham and Boyle, 2001; Iwase and Saito, 2004; Di Salvo et al., 2006; Figueroa et al., 2006; Barris and Button, 2008; Barros et al., 2011). More recently, new methods based on deep learning approaches, like convolutional neural networks, have improved the recognition and tracking of players in field sports, reducing the need of an operator to correct the tracking of players (Stein et al., 2017; Thomas et al., 2017; Cust et al., 2018; Renò et al., 2018). However, these methods all rely on multiple fixed cameras, and none have yet made use of a single drone camera. Concerning the use of drones in sports, Ferreira et al. (2015) and Karungaru et al. (2019) report that it is possible to detect and track players using a drone, but neither of these studies investigated its use for performance analysis. These studies also failed to validate the accuracy and reliability of the positional data obtained from the drone footage. Consequently, we believe that the current state of the art in computer vision and deep learning allows for tracking players automatically and provide positional data to derive performance indicators based on drone-based video technology (Thomas et al., 2017; Cust et al., 2018; Liang et al., 2019; Lee et al., 2020).

Thus, this study aims to describe a new method for position detection using a drone-based video system. We believe that the recent advancements in computer vision and deep learning can be used to reliably and automatically track players in a variety of sports settings. This study will be the first to provide validation of positional data obtained from drone footage in three different sports: tennis, Ultimate Frisbee, and soccer. This data will also be used to derive relevant performance indicators for each of these sports based on the drone-based video technology.

## 2 Materials and Methods

### 2.1 Sample

To collect representative, real-world data for tracking and for the validation of our drone-based video tracking system, we acquired three different samples with varying field sizes, field colors, number of players, and levels of expertise in three different sports: tennis, Ultimate Frisbee and soccer. GPS-and LPS-based technologies were used for the validation of our drone-based video tracking system. For tennis, the sample was represented by two 14-year-old male tennis players with eight and 9 years of experience, respectively. For Ultimate Frisbee, the sample data was collected during a trial match (n = 14), including current or former players from the German national team (age:28.35 ± 2.46 years). For soccer, eight female amateur soccer players (age:20.80 ± 0.83 years) participated in a small-sided game (4 vs 4).

All of the participants voluntarily gave informed consent to participate in the collection of spatiotemporal tracking data via drone technology. The data was anonymized to ensure confidentiality. All procedures performed in the study were in accordance with the Declaration of Helsinki.

### 2.2 Drones

An unmanned aerial vehicle is an aircraft without any human pilot, crew, or passengers on board. UAVs are a type of an unmanned aircraft system (UAS), which consist of an additional ground-based controller and a system of communication with the UAV (Abhishek et al., 2020). The drone used in this study was a Mavic Air 2 Model (SZ DJI Technology Co., Ltd. DJI) with Obstacle Sensing, Advanced Pilot Assistance 3.0, a fully stabilized 3-axis gimbal and 1/2″ sensor camera. Video frequency was set to 24 Hz with 4K resolution of 3.840 × 2.160 pixels. As the flight duration of this drone is around 34 min, we used a second drone of the same model to replace the first one and to ensure continuous data acquisition in case we exceeded this duration. The chosen height during stationary flight was determined based on the size of the field, weather conditions and was in accordance with the legal regulations for UAV, in our case the German regulations (www.gesetze-im-internet.de/luftvo_2015/). All of these variables were set to optimize the safety of the participants and the quality of the video footage through the unique bird’s-eye view perspective.

### 2.3 Data Acquisition

**FIGURE 1:**
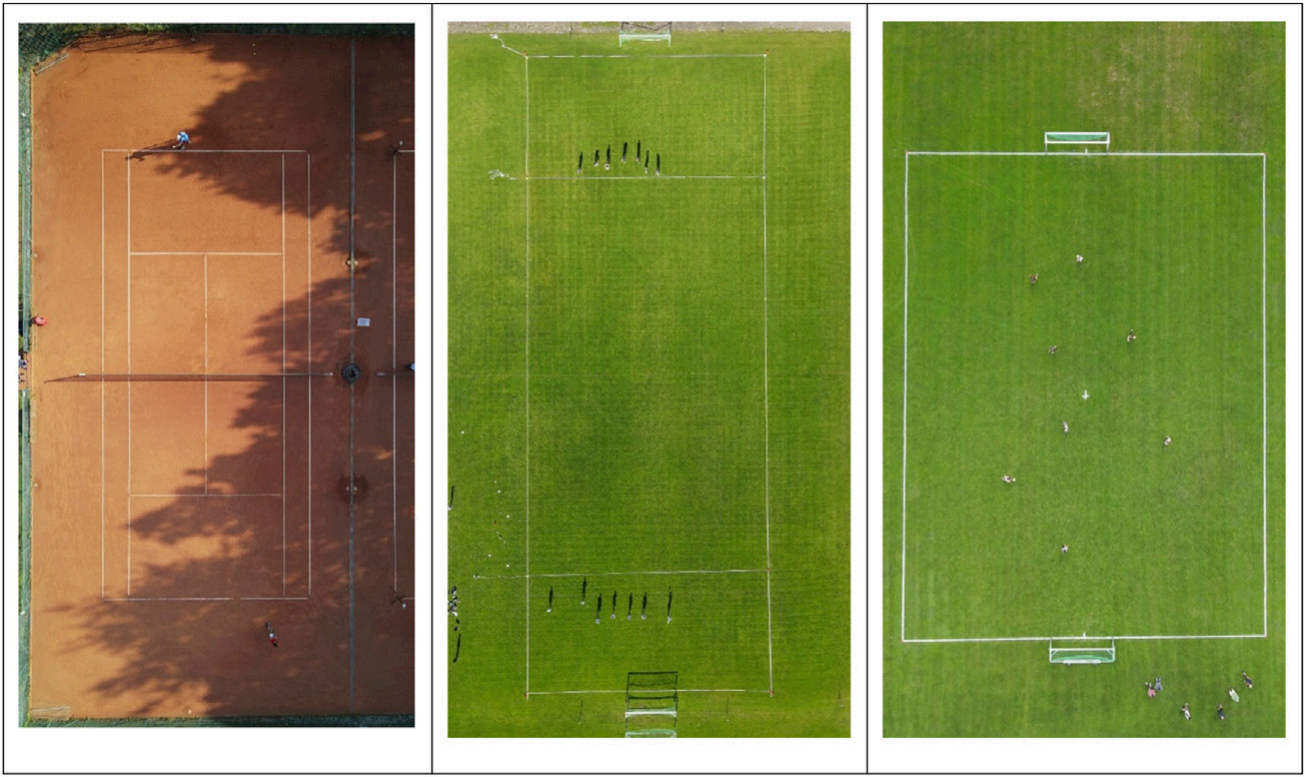
Drone perspective from a bird’s eye view for tennis (27 m height), Ultimate Frisbee (85 m height) and soccer (50 m height).

The data was collected in three different setups: on a tennis court (23.77 m × 8.23 m) with the drone hovering at a height of 27 m, on an Ultimate Frisbee field (97.11 m × 36.25 m) with the drone at a height of 85 m, and on a small-sided soccer field (39 m × 29 m) with the drone at height of 50 m. All three sports were recorded from a bird’s eye view with the drone positioned at the center of the court/field, enabling a full view of the field and the players, including the surrounding areas of interest, as shown in Figure 1.

Before each data acquisition, eight red cones (Ø = 0.15 m) were placed on the field, four of which were placed at the corners and the other four at the intersection lines (control points). For the Ultimate Frisbee setup, the control points were located where the end lines intersect with the side-lines in the intersections of the end line with the side lines. For the Tennis setup, the control points were located where the service lines intersect with the single side lines. The 2D locations of these cones (real-world coordinates) were measured using the tachymeter Trimble M3 Total Station with the Trimble Access software (Version: 2012.10). This system was used to measure the distance between a fixed point and the measurement device in X, Y, and Z coordinates. A reflective marker was placed according to the cones’ 2D center of mass (COM), which identified the target point with 0.002 m of accuracy.

The cones’ corresponding projections on the image (image plane coordinates) were digitized using our developed software (section 2.4). Thus, the homographic parameters of the mathematical image-object transformation were calculated, allowing for 2D kinematic analysis. This method for obtaining the transformation from 2D image coordinates to 2D object coordinates was based on 2D homography (Corke, 2017). Subsequently, both X and Y coordinates represent the transformed coordinates relative to the court/field coordinate system with origin in the bottom right of the field/court.

### 2.4 Tracking Algorithm

**FIGURE 2:**
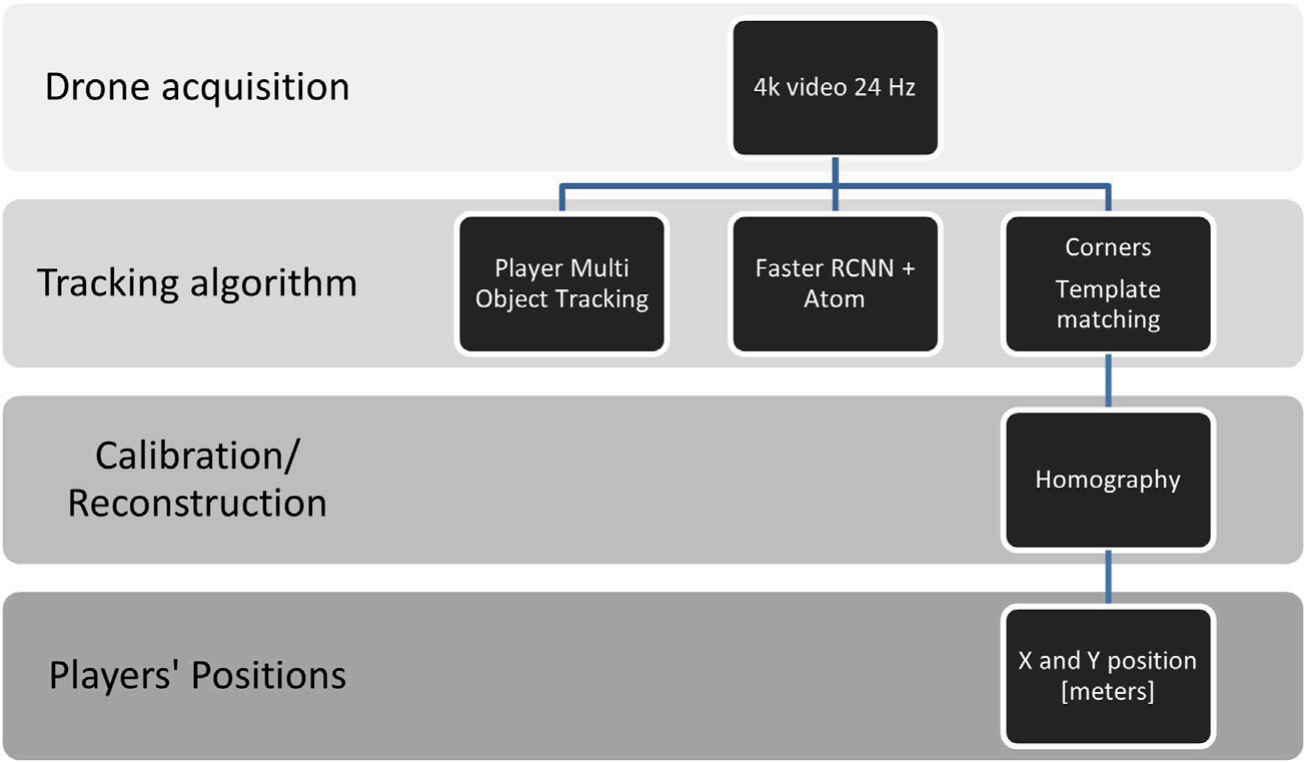
Block diagram showing the main steps of the proposed tracking method using a drone-based video system.

Tracking was done using a flexible software interface developed in the Python programming language (Python Software Foundation, https://www.python.org/). Figure 2 presents the block diagram of the tracking system, in which multiple object tracking was performed (Bewley et al., 2016; Milan et al., 2016). This was conducted with a 2 phase System. A Faster-RCNN object detection neural network was trained to recognize players from a bird’s eye view (Ren et al., 2015). Next, we tracked the initial players’ bounding boxes with a generic object tracker called Atom (Danelljan et al., 2019), which performs at the top of specific tracking benchmarks such as UAV123 and TrackingNet.

Errors in the tracking process of the bounding boxes were edited manually through a GUI written with QT (www.qt.io).

To transform the player coordinates from frame coordinates to real-world coordinates, we extracted multiple corners of the different game environments using template matching strategies. A personal computer (Intel(R) Core (TM) i7-7700HQ, CPU 2.80GHz, 16 GB RAM, Ubuntu) was used to track the players. All of the coordinates contained the X and Y coordinates of the players, and the corners were saved as a CSV file.

The X and Y positions of the players were defined as the center point of the bounding box enclosing the respective player’s outline. Following the tracking procedure, the X and Y positions of the players were reconstructed based on the four corner points extracted for calibration in MATLAB (R2020b, The MathWorks Inc., Natick, MA, United States) using 2D homography (Corke, 2017). Due to slight movements of the drone, the calibration was performed frame by frame to reduce errors.

### 2.5 Validation

To validate the drone-based video EPTS system developed in this work, two different tests were conducted. First, a static validation was performed using specific known and measured points on the court/field that were measured with the gold standard (tachymeter). Secondly, we conducted a dynamic validation. Ideally, this type of validation is conducted using 3D kinematic analysis like Vicon or Qualisys (Luteberget and Gilgien, 2020), but this is challenging and costly to do in field settings (Linke et al., 2018) and actually cannot be done in large environments like an Ultimate Frisbee field. The alternative is an approximation, which involves comparing the measurements the drone-based video system with other systems that have been reported in the literature (Frencken et al., 2010; Randers et al., 2010; Ogris et al., 2012; Varley et al., 2012; Buchheit et al., 2014; Ellens et al., 2021). In this case, we used a GPS system and a LPS system, described in Table 1.

**TABLE 1:** Description of the experimental design for tennis, Ultimate Frisbee and soccer, including the number of participants, gender, duration, match type, and tracking devices used.

\Sport	N	Gender	Overall duration (min)	Validation intervals	Exercise type	GPS	LPS	Drone
Tennis	2	Male	29	4 sessions	match	**✓**	**✓**	**✓**
Ultimate Frisbee	14	Mixed	34	3 sessions	match	**✓**		**✓**
Soccer	8	Female	5	1session	small-sided game	**✓**		**✓**

All participants in this study were equipped with at least one transponder for the GPS system (GPSports Sports Performance Indicator (SPI) Pro X, Canberra, Australia). For tennis, an additional transponder was attached for the LPS system (KINEXON Precision Technologies, Munich, Germany). The transponders were placed on the upper thoracic spine between the scapulae.

The GPS transponders were activated 15 min prior to data collection to allow for the acquisition of satellite signals, as only GPS signals that meet the internal quality thresholds established by the manufacturer are recorded (Shergill et al., 2021). The LPS transponders were activated at the same time to reduce contact between the investigators and the athletes in accordance with the COVID-19 guidelines at the time. Just before the start of a match, the drone was positioned above the center of the court/field and set to remain in a stationary position (see Figure 1).

#### 2.5.1 Static Validation

For the static validation measurements, the real-world coordinates of four cones on the field/pitch (Ø = 0.15 m) were measured with a tachymeter, which served as the gold standard. For the sake of comparison, 5 minutes of video were acquired at frequency of 24 Hz, just before the data collection session to compare the drone measurements with the tachymeter measurements on the tennis court and on the Ultimate Frisbee field.

Previous studies used a limited number of timepoints for one position to estimate the static measurement, for example by fixing a transponder to the ground for 2 minutes or by measuring the court/field manually before data acquisition (Lara et al., 2018; Linke et al., 2018).

#### 2.5.2 Dynamic Validation

Raw XY-positions from each of the EPTS were exported using the respective software (see Figure 2). The raw speed data was synchronized for speed using cross correlation. Which allowed for the calculation of the deviation between the GPS system and the drone system for each point in time and for each setting. Data from the systems was sampled at different frequencies: 15 Hz (GPS), 20 Hz (LPS) and 24 Hz (drone). All of the remaining data analysis steps were executed in MATLAB (R2020b, The MathWorks Inc., Natick, MA, United States). The data from the LPS system and the drone system was down-sampled to 15 Hz using a linear interpolation of the initial values. All positional data was filtered using a fourth-order Butterworth low pass frequency filter (Linke et al., 2018).

The dynamic validation was performed with two kinds of analysis. First, the cumulative distance measured by the drone system was compared to the distance from the LPS system for tennis and from the GPS system for all sports. Secondly, the distance covered across different speed zones was also compared: stationary walking (0–3.9 km/h), jogging (4.0–7.9 km/h), and quick running (above 8 km/h), mostly because the sample hardly reached speeds above 14 km/h. These speed zones were adapted from Krustrup and Mohr (2015).

Speed and acceleration data from the drone and LPS systems were derived from filtered positional data. The GPS system assesses speed data by the rate of change (Doppler) in the satellites’ electromagnetic signal frequency (Schutz and Herren, 2000). Therefore, the manufacturer’s speed variable was used and served as the basis to calculate acceleration.

### 2.6 Statistical Analysis

The accuracy of the static XY-position data was estimated by means of the root mean square error (RMSE) as seen in .

$$RMSE=\;\sqrt{\frac{\sum_{i=1}^{n}\left(x_{i}-y_{i}\right)²}{n}}$$ (1)

where *y*_*i*_ are the observations, *x*_*i*_ predicted values of a variable, and *n* the number of observations available for analysis.

Descriptive statistics are provided as means, standard deviations (SD) and coefficient of variation (CV). A Shapiro-Wilk test was used to test the normality of the data. In cases where the data failed the normality test, non-parametric test procedures were used to analyze the data (Wilcoxon signed-rank test).

To evaluate the performance of drone tracking compared to GPS and LPS systems in the three different sports contexts (tennis, Ultimate Frisbee, and soccer), a Bland-Altman plot was drawn to assess the level of systematic difference between measurements of the total distance covered by the players. Pearson’s correlations coefficients were classified as (small effect<0.3; medium <0.5; large >0.5). Reliability of total distance covered was assessed calculating intra-class correlation coefficients (ICC). ICC coefficients were classified according to Koo and Li (2016) into poor (ICC ≤0.5), moderate (ICC ≤0.75), good (ICC ≤0.9), and excellent (ICC >0.9). Statistical analyses were conducted in MATLAB R2020b (The MathWorks, Massachusetts, United States) and SPSS (v27.0.1.0).

## 3 Results

### 3.1 Static Validation

Table 2 shows the RMSE of the distances between the observed and expected positions on the court/field for the four control points used in the tennis match and in the Ultimate Frisbee match. It is important to reiterate that the four control points were placed in specific positions based on the different court/field sizes for tennis and Ultimate Frisbee.

**TABLE 2:** RMSE values for the four control points used to evaluate the static accuracy during the calibration procedure on the tennis court and on the Ultimate Frisbee field. Means and standard deviations are shown for both settings.

Control points	RMSE values (tennis)	RMSE values (Ultimate frisbee)
Number 1	0.04 m	0.20 m
Number 2	0.01 m	0.16 m
Number 3	0.04 m	0.11 m
Number 4	0.02 m	0.13 m
Mean ± sd	0.02 ± 0.01 m	0.15 ± 0.03 m

The mean RMSE for a static position on the tennis court was 0.02 m, 0.08% of the court’s length and 0.24% of the court’s width. For the Ultimate Frisbee field, the mean RMSE for a static position on the field was 0.15 m, 0.15% of the court’s length and 0.41% of the court’s width. The maximum RMSEs found in static positions on the tennis court and the Ultimate Frisbee field for a 5-min testing interval was 0.04 and 0.20 m, respectively.

### 3.2 Dynamic Validation

The regression analysis for the total distance between the drone and the GPS/LPS systems showed a significant linear regression (*p* < 0.05) for all three sports. For tennis, the *R* ^2^ value was 0.980 with a RMSE of 21.8 m, RMSE% of 5.78% for GPS, with an ICC value for consistency of 0.974 and the ICC for absolute agreement of 0.923 (*p* < 0.001); for LPS, the *R* ^2^ value was 0.999 with a RMSE of 5.1 m, RMSE% of 1.21%, with an ICC value for consistency of 0.999 and the ICC for absolute agreement of 0.998 (*p* < 0.001). For Ultimate Frisbee, the *R* ^2^ value was 0.996 with a RMSE of 10.7 m, RMSE% of 1.01%, with an ICC value for consistency of 0.998 and the ICC for absolute agreement of 0.984 (*p* < 0.001). For soccer, the *R* ^2^ value was 0.926 with a RMSE of 12.9 m, RMSE% of 3.11%, with an ICC value for consistency of 0.956 and the ICC for absolute agreement of 0.942 (*p* < 0.001).

Table 3 shows the descriptive statistics (means, standard deviations anc coefficient of variation) for the different tracking devices (Drone/GPS/LPS) regarding total distance covered and total distance covered in the three different speeds: stationary walking (0–3.9 km/h), jogging (4.0–7.9 km/h), quick running (above 8.0 km/h) in Tennis, Ultimate Frisbee (UF) and soccer small-sided game.

**TABLE 3:** Descriptive statistics for the different tracking devices (Drone/GPS/LPS) regarding total distance covered and total distance covered in the three different speeds: stationary walking (0–3.9 km/h), jogging (4.0–7.9 km/h), quick running (above 8.0 km/h) in Tennis, Ultimate Frisbee (UF) and Soccer small-sided game. Means, standard deviations and coefficient of variance are shown for all the settings.

	Device	Tennis			UF			Soccer		
		Mean	±SD	CV%	Mean	±SD	CV%	Mean	±SD	CV%
TOTAL DISTANCE (M)	Drone	430.6	168.6	39.15	1022.2	166.9	16.32	404.2	49.0	12.12
	GPS	377.1	141.0	37.39	1050.9	167.9	15.97	413.5	43.8	10.59
	LPS	420.0	166.2	39.57	—	—	—	—	—	—
DISTANCE IN SPEED	Drone	143.5	60.9	42.43	148.6	28.0	18.84	90.5	15.7	17.34
0–3.9 kM/H (M)	GPS	205.7	87.7	42.63	143.2	25.3	17.66	85.2	12.9	15.14
	LPS	128.17	57.1	44.55	—	—	—	—	—	—
DISTANCE IN SPEED	Drone	217.8	104.0	47.75	297.2	54.0	18.16	168.4	35.9	21.31
4.0–7.9 kM/H (M)	GPS	143.4	60.9	42.46	303.3	56.8	18.72	161.4	27.6	17.10
	LPS	220.4	101.3	45.96	—	—	—	—	—	—
DISTANCE IN SPEED	Drone	69.3	31.1	44.87	576.4	180.0	31.22	145.2	47.2	32.50
>8.0 kM/H (M)	GPS	27.9	13.8	49.46	684.37	183.0	26.73	166.9	57.3	34.33
	LPS	71.4	34.6	48.45	—	—	—	—	—	—

**FIGURE 3:**
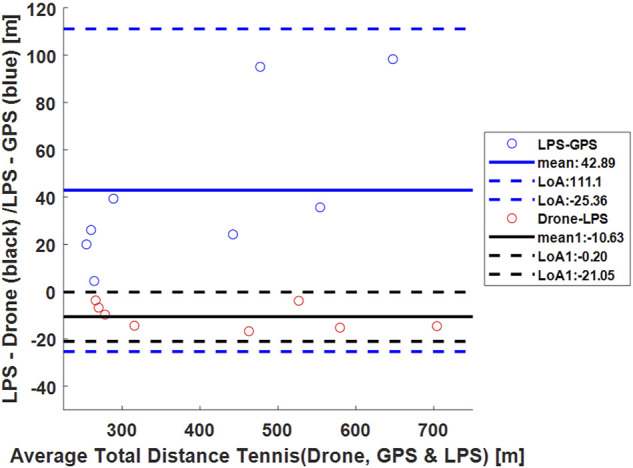
Bland-Altman plot for the total distance covered in a tennis match measured by the drone, GPS, and LPS. Dashed blue lines show the limits of agreement (111.10 m and -25.36 m), and the continuous line shows the mean (42.89 m) between GPS and LPS. Dashed black lines show the limits of agreement (−0.20 m and −21.05 m), and the continuous line shows the mean (−10.63 m) between the drone and LPS.

**FIGURE 4:**
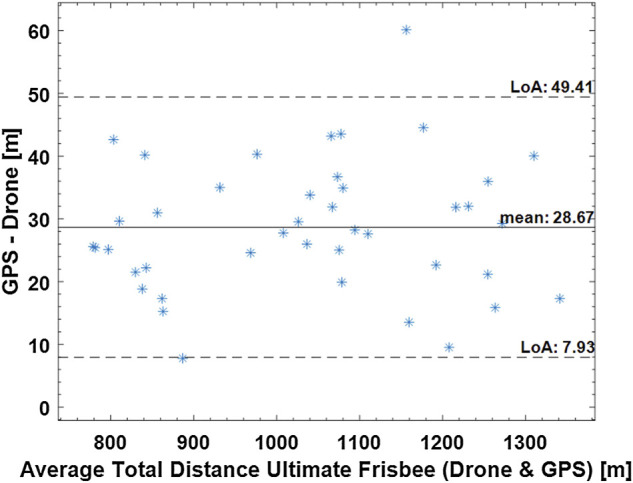
Bland-Altman plot for the total distance covered in an Ultimate Frisbee match measured by the drone and GPS. Dashed lines show the limits of agreement (7.93 and 49.41), and the continuous line shows the mean 28.87 m.

**FIGURE 5:**
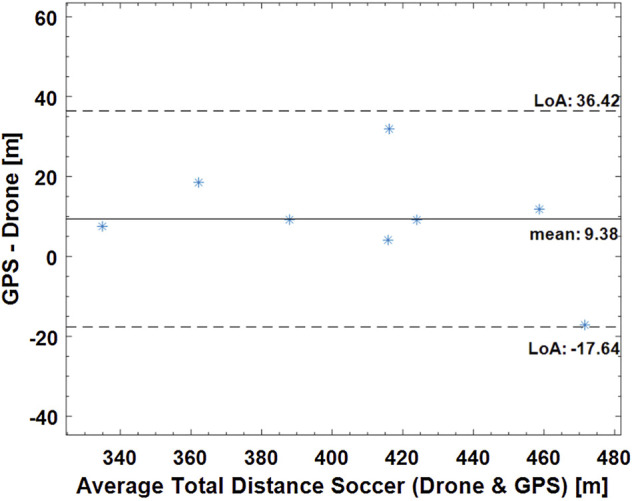
Bland-Altman plot for the total distance covered in a small-sided game soccer match measured by the drone and GPS. Dashed lines show the limits of agreement (36.42 m and −17.64 m), and the continuous line shows the mean (9.39 m).

Figures 3–5 show Bland-Altman plots with the mean values between the measurements and the lower and upper limits of agreement for the total distance in all three sports (tennis, Ultimate Frisbee, and soccer).

Regarding the total distance covered, an absolute difference of 13.67% was calculated for tennis, 2.78% for the Ultimate Frisbee, and 2.36% for soccer between the drone and the GPS. The error between GPS and LPS was 9.42% in the tennis match. The total distance covered between the drone and LPS had an absolute difference of 2.68% in the tennis match.

**FIGURE 6:**
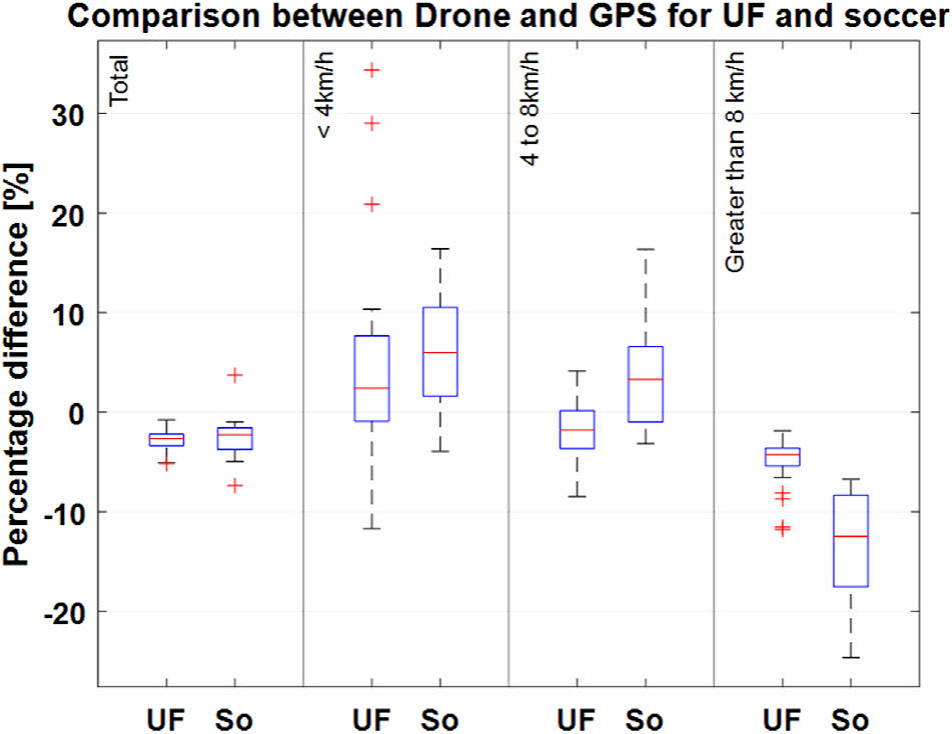
Percentage deviation of the total distance measurements between the drone-based video system and GPS in Ultimate and soccer for different speed zones. Boxplots show the respective median (red line); the bottom and top edges of the box indicate the 25th and 75th percentiles (blue box). The whiskers extend to the most extreme data points without considering any outliers (“+” symbol). UF (Ultimate Frisbee), So (soccer small-sided game).

**FIGURE 7:**
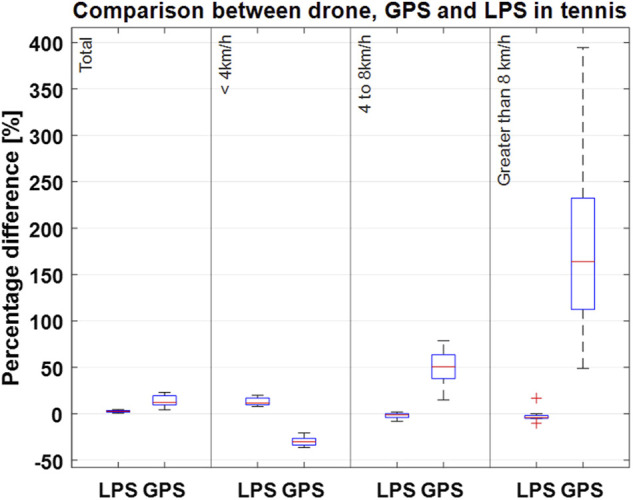
Percentage deviation of the total distance measurements between the drone-based video system and LPS and drone-based video system and GPS in tennis for different speed zones. Boxplots show the respective median (red line); the bottom and top edges of the box indicate the 25th and 75th percentiles (blue box). The whiskers extend to the most extreme data points without considering any outliers (“+” symbol).

Figures 6, 7 show the deviation in the covered distances in total and at different speeds, as illustrated by box plots. For Ultimate Frisbee and soccer, Figure 6 shows the measurements from the drone and GPS. For tennis, Figure 7 shows the measurements from the drone, GPS, and LPS. Since the players in this sample hardly ever reached speeds above 14 km/h, the distances covered in each speed zones were presented as follows: up to 4 km/h, from 4 to 8 km/h and greater than 8 km/h.

### 3.3 Exemplary Results in Sports

**FIGURE 8:**
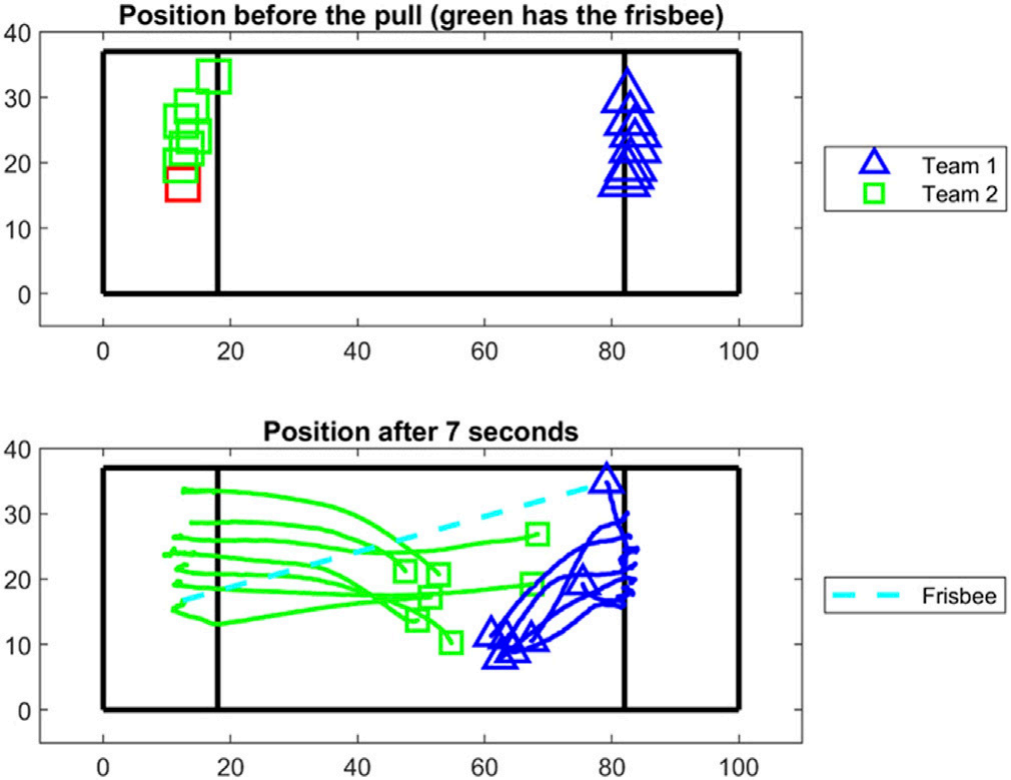
Tracking data recorded with a drone from an Ultimate Frisbee game during the pull and 7 s afterwards. Team 2 (red square) has the frisbee (upper field). Frisbee was not tracked; blue dashed line represents the vector from starting to end position of the passe.

**FIGURE 9:**
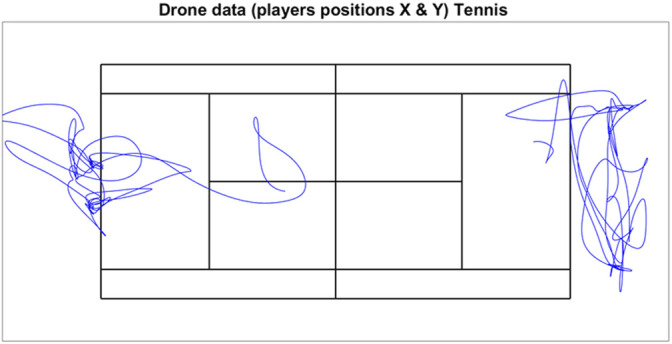
Example of X and Y positions obtained by the drone system for two players during 1-min of gameplay in a tennis match.

**FIGURE 10:**
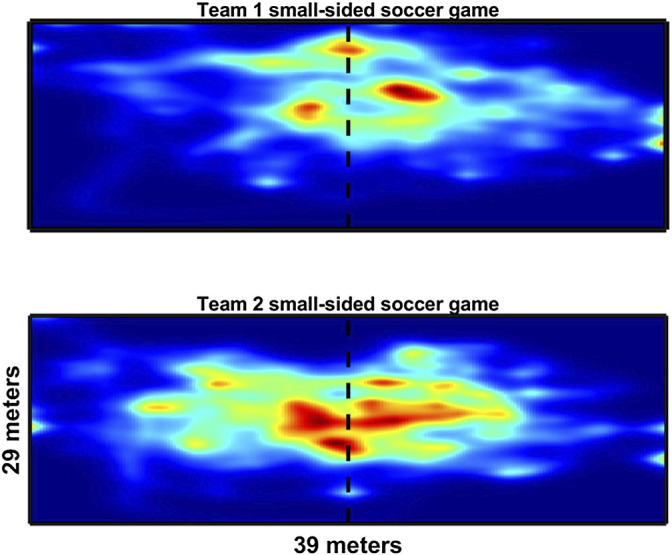
Heatmap representation of the two teams (4 vs 4) during 5 min of gameplay in a small-sided soccer game.

Figures 8–10 show exemplary results in the three different sports that can be obtained from a drone-based video EPTS system. Figure 8 shows the typical movements of players (n = 14) during the pull in an Ultimate Frisbee game, showing that the drone system can deliver not only X and Y positions of the players, but also allows for new insights about tactical displacement using the bird’s eye view. Figure 9 illustrates the X and Y positions of tennis players on the court during a match. Figure 10 is a direct application of tracking data for game analysis in small-sided soccer games based on heatmaps.

## 4 Discussion

This study is the first to demonstrate the application of a drone-based video system for the performance analysis of three different sports: tennis, Ultimate Frisbee, and soccer. The results not only show the system’s ability to detect and track players from a bird’s eye view (Ferreira et al., 2015; Karungaru et al., 2019), but also to collect and generate position detection data. Furthermore, the results from this study are validated against some of the existing position detection technologies (GPS and LPS) that are currently used in performance analysis.

The mean measurement error found for the static validation was less than 0.41% of the size of the court/field for all three sport settings. The maximum difference found between the known and measured positions on the tennis court and on the Ultimate Frisbee field was 0.04 and 0.20 m, respectively. These values are lower than the ones reported by Alcock et al. (2009), who reported mean errors of 1.5% of the width and 2.5% of the length of a soccer field. The results from the static validation support the accuracy of the drone-based video system for the measurement of static positions on the court/field when compared to the gold standard (the tachymeter). This improved accuracy may be explained by the fact that every frame from the drone footage is calibrated individually since the drone is subjected to small movements during flight.

For the dynamic validation, the measurements of the total distance covered, and the distances covered in different speed zones were compared between the drone system and commercial GPS and LPS systems. The total distance covered measured by the drone had a high correlation with both the GPS and LPS systems, with Pearson correlation coefficients of 0.96 and 0.99, respectively. It is important to clarify that correlation, in this case, does not mean that all of the systems came to the same measurement, but that the systems are related to each other. A better way to evaluate the agreement between the different methods might be a regression analysis. In this way, we would need to determine a formula that best predicts the magnitude of a value obtained from the drone as it relates to another measuring device (GPS or LPS).

Regression analysis shows a *R* ^2^ value higher than 0.90, and ICC results showed excellent consistency and absolute agreement in the measurement of the total distance covered. Buchheit et al. (2014) report small differences (5.4%) between GPS and optical tracking systems in relation to total distance covered. In this study, the differences in the total distance covered between the drone and GPS systems are around 3% for Ultimate Frisbee and soccer small-sided game.

However, at this time, there is no gold standard used for dynamic validation of drone-based position detection. The authors chose to present Bland–Altman plots that illustrate some qualitative data, such as the mean bias (how much does the drone deviate from the measurements obtained by the GPS and/or LPS) and the confidence intervals, that may be used to explain some of the systematic and random deviations observed between the different tracking technologies in this study.

The limits of agreement in the Bland-Altmann plot for total distances are 49.41 and 7.99 m for Ultimate Frisbee (drone vs GPS; see Figure 4), 36.52 m and −17.64 m for soccer (drone vs GPS; see Figure 5), 15.74 m and −122 m for tennis (drone vs GPS) and −0.26 m and −21 m for tennis (drone vs LPS; see Figure 6). The limits of agreement for the drone vs LPS in tennis look better compared to the results for GPS, as the size of the field may have hindered the precision of the GPS measurements.

Overall, there was excellent agreement in the measured distances covered in different speed zones during the tennis match between the drone and LPS systems. However, there were some noteworthy differences between these two systems at higher speeds (above 8 km/h), which suggests there might have been a systematic error during data collection. For validation purposes, it would be ideal to compare the drone system to an accepted gold standard as the reference system to confirm the accuracy of instantaneous position, speed, and acceleration values. This type of validation should be conducted in the near future for the drone-based video system, especially using regression analysis to compare the results against other EPTS or gold standards like Vicon or Qualisys (Luteberget and Gilgien, 2020).

Based on the findings of this study, the application of a drone-based video system resulted in more accurate static positions and dynamic trajectories (with less deviation) compared to LPS- and GPS-based systems. This finding is in line with previous studies that compared traditional video-based systems with GPS-based systems (Buchheit et al., 2014; Linke et al., 2018). While it appears that video-based systems generate more accurate and representative results for multi-player tracking compared to sensor-based systems, the process still requires supervision by an experienced operator, as the player trajectories can be unpredictable. Nevertheless, the advantages of a drone-based video system also include video footage from a bird’s eye view, which allows for a unique perspective for tactical analysis of both one’s own team and the opposing team. The drone’s main advantage is its versatility, as it can be used in training or during competitions without the need to install any additional equipment (traditional video-based systems) or attach any devices to the players (sensor-based systems). A drone-based video system also provides a different vantage point than traditional video-based systems, as the drone can fly above the court/field and be maneuvered to remain in a stationary position. Lastly, drones are accessible and less costly than other EPTS, facilitating the ability to use position detection methods for performance analysis at all levels.

It is worth mentioning that the current work presents some limitations regarding its validation at higher speeds, greater than 8 km/h, given that the study sample did not reach such speeds. Nevertheless, the results found in this study are of sufficient validity for Ultimate Frisbee, tennis, and small-sided games in soccer, where other authors have also reported that higher speeds are rarely reached (Linke et al., 2018; Linke et al., 2020).

## 5 Conclusion

To the best of our knowledge, this is the first study to demonstrate and validate the use of drones for performance analysis, as well as present examples of their application in several different game sports (tennis, Ultimate Frisbee, and soccer). The drone-based video system not only detects and tracks players’ positions and trajectories, but also provides performance analysis metrics in competition and training settings. The results were validated against known position detection technologies on the market (GPS and LPS). By implementing a drone-based video system, coaches and performance analysts will be able to visualize and quantify the X and Y positions of all players on the court/field. Furthermore, the drone footage will allow for conclusions about the physical demands and tactical behaviors observed in training and in competition across a variety of game sports. Future research can build upon the findings of this work by further testing the drone-based video system in different sport contexts and environments, such as indoor use. In the meantime, this study has shown that drone-based video position detection is both feasible and reliable; this technology has the potential to enhance performance analysis in sports and facilitate access to position detection methods.

## Data Availability

The raw data supporting the conclusions of this article will be made available by the authors, without undue reservation.
